# ATFS-1 plays no repressive role in the regulation of epidermal immune response

**DOI:** 10.17912/micropub.biology.000525

**Published:** 2022-02-22

**Authors:** Celine N Martineau, Claire A Maynard, Nathalie Pujol

**Affiliations:** 1 Aix Marseille Univ, INSERM, CNRS, CIML, Turing Centre for Living Systems, Marseille, France

## Abstract

Fungal infection triggers the induction of antimicrobial peptide (AMP) genes in the epidermis (Pujol et al, 2008). We previously showed that this effect is suppressed by the mitochondrial unfolded protein response (UPR^mt^), which can be activated by knockdown of select genes including the mitochondrial metalloprotease *spg-7* (Zugasti et al, 2016). Here, we confirm that RNAi against *spg-7* triggers the UPR^mt ^and blocks AMP induction during infection, whereas infection itself does not trigger the UPR^mt^. ATFS-1 is a key factor in the UPR^mt^, mediating much of the associated transcriptional response. We find that, surprisingly, ATFS-1 is not required for the suppression of AMP induction provoked by *spg-7(RNAi)*. These data show that the mitochondrial dysfunction that blocks the immune response upon infection or wounding is independent of ATFS-1.

**Figure 1. ATFS-1 is not required for the induction of the epidermal immune response, nor for its inhibition:**
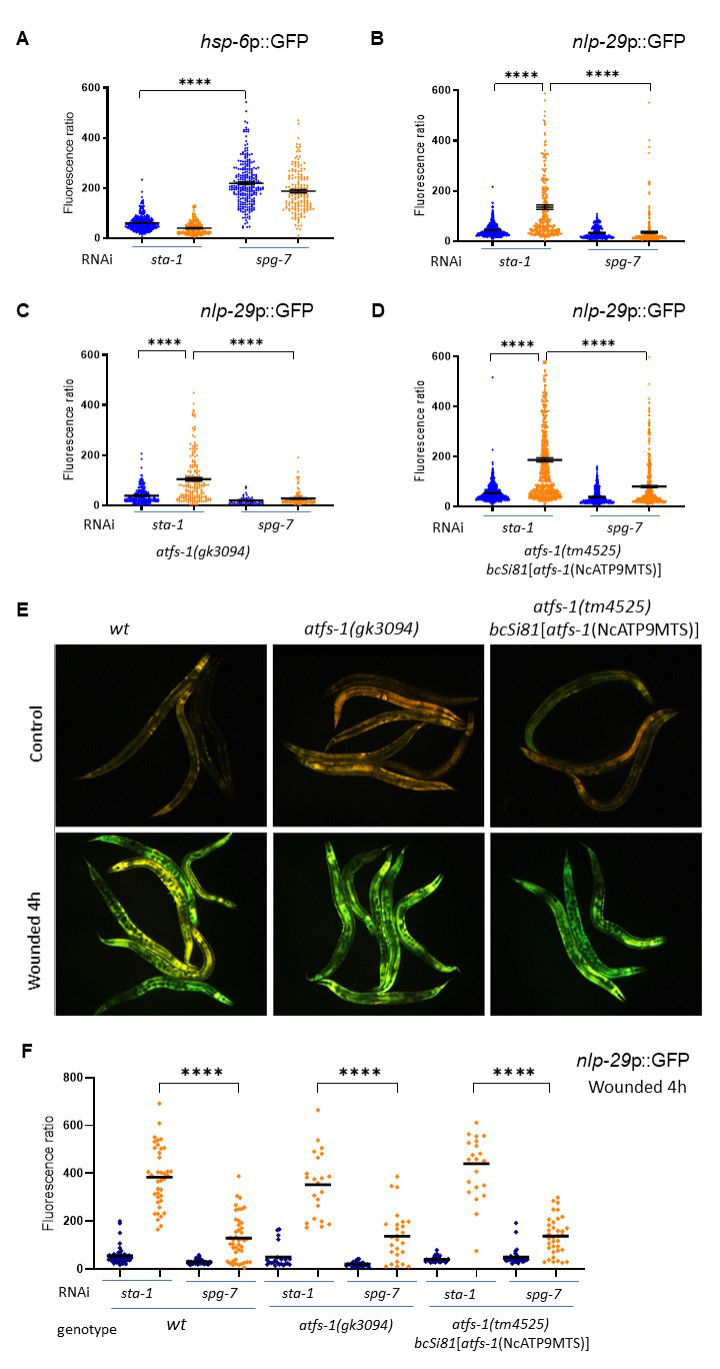
(A-D) Quantification of relative green fluorescence (GFP/size) in young adult worms after infection (orange) or not (blue) withthe fungus *Drechmeria coniospora* treated with the either *sta-1* (control) or *spg-7* RNAi clones, with mean and SEM, **** p<0.0001. The induction of immune response was followed with a *nlp-29*p::GFP reporter using the *frIs7* transgene (A,C & D). The induction of UPR^mt^ was followed with a *hsp-6*p::GFP reporter using the *zcIs13* transgene(B). (A-B) Compared to *sta-1* control clone, the inactivation of *spg-7* induces the UPR^mt^ (B) and blocks the induction of *nlp-29p*::GFP upon infection in the wild type (A), as previously shown (Zugasti *et al.*, 2014). (C-D) The same results were obtained in *atfs-1(gk3094)* mutant background (C) or in the background of an *atfs-1(tm4525)* mutant rescued with a form of ATFS-1 engineered to include a strong MTS signal (*bcSi81[atfs-1(NcATP9MTS)]*) (Rolland *et al.*, 2019) (D). (E) Representatives images of the induction of immune response upon wounding followed with the same *frIs7* reporter transgene. The induction of *nlp-29*p::GFP reporter is visualised simultanously with the constitutive expression of *col-12*p::RFP reporter in the epidermis with a GFP long pass filter. (F) Quantification of relative green fluorescence (GFP/size) in young adult worms after wounding (orange) or not (blue)treated with the either *sta-1* (control) or *spg-7* RNAi clones, with mean and SEM, **** p<0.0001.

## Description

*Drechmeria coniospora* is a natural fungal pathogen of *Caenorhabditis elegans*. Fungal spores are able to pierce the cuticle of the worm, leading to hyphal growth in the entire organism. *C. elegans* counters the infection by triggering a rapid innate immune response. A hallmark of the response is the secretion of antimicrobial peptides (AMP) encoded by the *cnc* (caenacin) family and certain *nlp* (neuro-peptide-like protein) genes (Dierking *et al.*, 2011; Taffoni *et al.*, 2020). We previously showed that impairing mitochondrial function and inducing the mitochondrial unfolded protein response (UPR^mt^), blocks the expression of AMP genes after infection by *D. coniospora*, possibly through cross-tissue signaling between the intestine and the epidermis (Zugasti *et al.*, 2016; Ewbank & Pujol, 2016). The molecular pathways involved, however, remain unknown.

Here, we first confirmed that knock-down of the mitochondrial metalloprotease *spg-7* by RNAi activates the UPRmt through the induction of *hsp-6p*::GFP reporter (Figure 1A) (Yoneda *et al.*, 2004), and showed that infection by *D. coniospora* did not induce this reporter. Using a transcriptional reporter strain for AMP expression (*nlp-29*p::GFP) (Pujol *et al.*, 2008), we also confirmed that *spg-7* inactivation resulted in a robust block of AMP expression after infection compared to the *sta-1* control clone (Figure 1B) (Zugasti *et al.*, 2016).

We then investigated whether ATFS-1, a master regulator of the UPR^mt^, is required for the *spg-7* suppression. As the responsiveness of ATFS-1 is dependent on its relatively weak mitochondrial targeting sequence (Rolland *et al.*, 2019), its function can be impaired not only by loss-of function mutations but also by alterations that strengthen the mitochondrial targeting sequence (MTS). To examine AMP expression under both of these conditions, we crossed the *nlp-29p*::GFP reporter to the *atfs-1(gk3094)* loss of function mutant and to the *atfs-1(tm4525)* loss of function mutant expressing an allele with a strong MTS (*bcSi81[atfs-1(NcATP9MTS)]*), known to be incapable of triggering the normal UPR^mt^-associated transcriptional response (Rolland *et al.*, 2019) and assayed the level of suppression of *nlp-29*p::GFP expression in response to *spg-7(RNAi)*. In our hands, loss of *atfs-1* did not change the induction of *nlp-29* expression associated with infection by *D. coniospora*. Further, there was a clear suppression of AMP reporter gene expression in both mutant backgrounds upon *spg-7(RNAi)* following infection (Figures 1C and 1D). We also showed that *atfs-1* is not required for the induction of the same immune response upon wounding (Figure 1E), nor for its suppression by *spg-7* RNAi (Figure 1F). We conclude that *atfs-1* is not required for the normal epidermal response to fungal infection or wounding and that the signal that provokes the repression of *nlp-29* expression upon mitochondrial dysfunction is independent of the key UPR^mt^ transcription factor ATFS-1.

## Methods

**RNA interference**

RNAi bacterial clones were obtained from the Ahringer library and verified by sequencing (Kamath *et al.*, 2003). RNAi bacteria were seeded on NGM plates supplemented with 100 μg/ml ampicillin and 1 mM Isopropyl-β-D-thiogalactopyranoside (IPTG). Worms were transferred onto RNAi plates as L3 larvae to avoid the lethality associated with *spg-7*(RNAi) and cultured at 25 °C until young adult stage.

***D. coniospora* infection & wounding**

Infections with *D. coniospora* were carried out at 25 °C as described (Pujol *et al.*, 2008). Briefly, synchronized L4 worms obtained following treatment with an alkaline hypochlorite solution, were infected by adding 100 μl of a fresh spore solution to RNAi plates and then incubated at 25 °C for 18h. Wounding was performed with a microinjection needle in the tail region of Day1 adult worms and analysed after 4h at 25 °C as described (Taffoni *et al.*, 2020).

**Analyses with the Biosort worm sorter**

Fluorescent protein expression of reporter strains was quantified with the COPAS (Complex Object Parametric Analyzer and Sorter) Biosort system (Union Biometrica; Holliston, MA) as described (Labed *et al.*, 2008). For each strain, a minimum of 50 synchronized young adult worms were analyzed for length (assessed as TOF, time of flight), optical density (assessed as extinction) and Green and/or Red fluorescence (GFP/Red). Raw data were filtered on the TOF for adult worms (400 ≤ TOF ≤ 1000). Fluorescent ratio (Green/TOF) is presented for each worm with mean and SEM for each conditions. Statistical analyses were performed in Graphpad Prism software using one-way ANOVA with Bonferroni correction.

## Reagents

IG274 *frIs7[nlp-29p::GFP, col-12p::DsRed] IV* (Pujol *et al.*, 2008)

IG1825 *atfs-1(gk3094) V; frIs7[nlp-29p::GFP, col-12p::DsRed] IV* (This study)

IG1830 *bcSi81[atfs-1(NcATP9MTS)] II; frIs7[nlp-29p::GFP, col-12p::DsRed] IV; atfs-1(tm4525) zcIs9 [hsp-60p::GFP] V* (This study)

SJ4100 *zcIs13[hsp-6::GFP] V* (Yoneda *et al.*, 2004)

MD4323 *bcSi81[atfs-1(NcATP9MTS)] II; unc-119(ed3) III; atfs-1(tm4525) zcIs9[hsp-60p::GFP] V* (Rolland *et al.*, 2019)

VC3201 *atfs-1(gk3094) V (C. elegans* Deletion Mutant Consortium, 2012)

*spg-7* RNAi clone sjj_Y47G6A_247.f

*sta-1* RNAi clone sjj_Y51H4A.o
